# Money, Economic Abuse, and Food Insecurity: A Qualitative Study among Young Nigerian Women with a History of Intimate Partner Violence

**DOI:** 10.3390/adolescents3020023

**Published:** 2023-05-25

**Authors:** Elizabeth L. Frost, Olufunmilayo I. Fawole, Omowumi O. Okedare, Mobolaji M. Salawu, Susan M. Kiene, Camarina Augusto, Elizabeth Reed

**Affiliations:** 1 School of Public Health, San Diego State University, San Diego, CA 92182, USA; 2 Department of Epidemiology and Medical Statistics, Faculty of Public Health, College of Medicine, University of Ibadan, Ibadan 200212, Nigeria

**Keywords:** intimate partner violence, economic vulnerability, food insecurity, economic abuse

## Abstract

Intimate partner violence (IPV) occurs in high proportions among young women, with long-lasting adverse health and social outcomes. Recent research findings suggest that experiencing economic vulnerability may influence the ways in which young women experience or are at risk for IPV, including economic abuse. Economic abuse, a form of IPV, involves a partner’s control over money and other economic resources or activities. This study explored economic vulnerability and IPV, including economic abuse, among young Nigerian women reporting a recent history of IPV. In-depth interviews (n = 25) were conducted with women aged 18–30 years who had experienced IPV in the past year. Women were recruited from community and health facilities in low-income neighborhoods of Ibadan, Nigeria. A semi-structured interview guide was used to gather data on women’s economic vulnerability (e.g., food security, living situation, employment/education opportunities, family financial support, economic independence) and experiences of IPV. An analysis was conducted using a thematic analysis approach. The coding scheme was based on interview protocols, adding open codes from emergent themes identified in the interviews. On average, participants were 21 years old, most had children (68%) and reported to be cohabitating with a male partner (56%), and 48% had less than a secondary level of education. Among the emergent themes, women reported economic vulnerability as being financially dependent on a male partner for basic needs. Among this sample, economic vulnerability was exacerbated by limited education, training, and work opportunities, and a disproportionate burden of household labor. Economic vulnerability precipitated all forms of IPV, including economic abuse, as well as sexual and pregnancy coercion. Economic abuse was reported to occur when male partners controlled household finances and denied women adequate allowance to purchase household food, including food for children. Findings from this qualitative study suggest that interventions promoting educational and employment opportunities may be critical to reducing financial reliance on male partners and young women’s vulnerability to economic abuse and other forms of IPV. More research and programmatic work are needed on food deprivation as a form of economic abuse affecting women and their children.

## 1. Introduction

Intimate partner violence (IPV) is a major global public health problem, contributing to significant morbidity and mortality worldwide [1]. Exposure to IPV has been documented as the third leading cause of mortality among women of reproductive age [2]. IPV is associated with multiple mental, physical, and behavioral poor health outcomes, including depression, anxiety, substance use, HIV/STIs, and adolescent pregnancy [2–5]. Globally, almost 30% of all women who have been in a relationship have experienced physical and/or sexual violence by an intimate partner [6].

A less well-known form of IPV is economic abuse, defined as a male partner’s control over money and other economic resources or activities, and control in deciding how household finances should be spent [7]. Economic abuse, also known as financial abuse or economic violence, is a term that has not been previously well-defined or measured in the literature, particularly due to the challenges in separating behaviors of economic abuse from women’s experiences of economic insecurity [8]. Various forms of economic abuse that have been reported include placing women on strict allowances, forcing them to beg for money, and denying or restricting women access to basic needs such as food, clothing, or healthcare. Economic abuse has also been reported to include refusal by the male partner to contribute to the household income, denying a woman from commencing or completing her education, vocational training, or finding employment, and limiting a woman’s access to cash or credit facilities.

IPV, including economic abuse, stems from traditional gender norms that support male dominance in heterosexual relationships. These same norms also devalue the prioritization of girls’ education, and restrict employment opportunities for young women [9]. In addition, compared to men, financial resources such as a bank account, savings account, or loans remain inaccessible to many women, but even more so for young women or girls from poor communities across the globe [10]. These gender-based structural inequities in education, access to the labor market, and access to financial resources create situations where women are less likely to be financially independent compared to men. In turn, young women may seek intimate or sexual relationships with male partners in order to obtain financial security [11,12], which places women at risk of becoming financially dependent on male partnerships. Dependency on a male partner for financial support can result in a power imbalance within the relationship that reduces a woman’s decision-making ability and her control over financially related household decisions, both of which increases the risk for all forms of IPV (e.g., physical, sexual coercion and violence, controlling behaviors, manipulation, isolation, and economic abuse). Economic abuse further reinforces women’s dependency on a male partner for money to meet their personal needs and the needs of their children [7,13]. Reliance on a male partner also makes it challenging for women to leave abusive relationships [14–16].

Throughout most of sub-Saharan African, physical and sexual IPV is widespread. In Nigeria, the prevalence of IPV is of notable concern. A study in Southwest Nigeria among Igbo migrant women aged 15–49 years old documented an estimated lifetime prevalence of IPV as high as 87%, while the prevalence of IPV in the past 12 months was 20% [17]. Similarly, among female university students in Ibadan, Nigeria, the lifetime prevalence of IPV was reported to be 42% [5]. While under-reporting experiences of IPV is an issue among women across the globe, it may be more pronounced in Nigeria, where challenges to reporting IPV include inaction of police, stigma associated with divorce or separation, fear of male partner retribution, loss of shelter, fear of impact on the children, and community norms that justify wife-beating [18]. When surveyed, 84% of Nigerian women did not want to report an IPV incident, with close to half of the women citing a need to protect their marriage as the main reason [19].

While previous studies shed light on the high prevalence of IPV in Nigeria and the negative health consequences, very few studies have sought to understand how economic abuse is occurring and the specific scenarios in which it takes place, particularly among young Nigerian women [20]. Understanding young women’s vulnerability to IPV, specifically economic abuse, is imperative for developing further education and other programmatic initiatives to negate the consequences of gender-based violence and support girls to have healthy relationships. More research is needed to understand the context and specific scenarios associated with young women’s experiences of economic abuse. Furthermore, the majority of previous studies on economic vulnerability and economic abuse recruited participants from a university setting or healthcare facilities [5,16,21–23] leaving a gap in understanding the situation of a younger population of women and girls, including those from low-income communities and who may be at highest risk for economic abuse.

The objective of our study was to explore economic vulnerability and IPV, including economic abuse, among young Nigerian women reporting recent IPV. This study complements previous work conducted among young Nigerian women in its use of qualitative data to understand the intersection of economic vulnerability and all forms of IPV, including economic abuse [5,7,20,24].

## 2. Materials and Methods

Data for this study were drawn from qualitative in-depth interviews among a sample of young Nigerian women aged 18–30 years who had a history of experiencing IPV. A total of 25 women were recruited from community organizations and health facilities in low-income neighborhoods of Ibadan. A thematic analysis approach was used to analyze the in-depth interviews, while focusing on themes that address the intersection of economic vulnerability and all forms of IPV. Based on this study’s research objective, and because there have been few previous studies on economic abuse in Nigeria, a thematic analysis was selected as the best approach to generate themes across the interviews regarding the ways in which young women are experiencing economic vulnerability, and how these influence IPV, including economic abuse.

### 2.1. Study Setting and Sample

This study was conducted in Ibadan, Oyo State, Southwest Nigeria. Ibadan is the third largest Nigerian city, with an estimated growth rate of 2.5%. In 2019, Ibadan had a population of 3,552,000, an increase from the 2006 population estimates of 2,567,000 [25]. The economy in Ibadan consists mainly of craft and trade, with the majority of women primarily partaking in trading activities. For this study, young women were recruited from community and health facilities located in low-income communities in Ibadan, Nigeria, where IPV has been reported in high proportions.

Eligibility criteria were based on age and previous experience of IPV. Eligible participants were women between 18 and 30 years of age who reported having a history of IPV at least once in the past year prior to the study. Participants were screened via a questionnaire to ensure they were eligible for the study. The sample represented various age groups, educational levels, and other social categories, including whether the women had children. The short screening questionnaire took approximately 5 to 10 minutes and was completely voluntary. The screening was conducted in a private location and facilitated by research staff trained in implementing sensitive questions regarding IPV. To assess eligibility, IPV was measured using physical and sexual IPV items from the World Health Organization (WHO) multi-country violence against women study [26]. Physical violence was measured by participants reporting if a male partner had slapped, punched, pushed, kicked, or choked them, as well as being threatened with or had a gun, knife, or weapon used against them. Sexual coercion was measured by participants reporting if a male partner had forced them into having sexual intercourse, using threats or intimidation to get them to have sex, making participants perform sexual acts (other than intercourse) against their wishes, or using physical force (such as holding them down or hitting them) to have sex with them. Psychological violence was measured by participants reporting if a male partner had insulted, belittled, humiliated, or threatened to beat them. The reason for refusal to participate or ineligibility was recorded. All women who participated in the brief questionnaire received a small reimbursement gift including a hand towel and/or soap.

### 2.2. Procedures

Trained and experienced bilingual (Yoruba-English) facilitators conducted informed consent among all participants, and subsequently implemented the semi-structured in-depth interviews. Interviews, lasting about 60 minutes, took place in a private room provided by the respondent, and were audio recorded. Interviews conducted in Yoruba were translated and transcribed into English by two independent members of the research team, and then back translated into Yoruba to ensure that the original meanings of the participants’ responses were maintained. Participants were reimbursed NGN 3600 (USD 10) upon completion of the interview. Ethical approval was obtained from the University of Ibadan/University College Hospital Joint Institutional Review Board and the San Diego State University Human Research Protection Program.

### 2.3. Measures

Participant’s socio-demographic data, such as age, marital status, and highest level of education, were collected via the eligibility screener and used to characterize the sample. A semi-structured interview guide was used, and included themes identified via the existing empirical literature and from previous work conducted by one of the authors regarding the role of economic vulnerability in influencing IPV [27]. Participants were asked open-ended questions with probes, allowing for a discussion between the interviewer and the interviewee. Topics of discussion included their living situation, financial situation, financial decision-making power in relationships, economic stability/independence, financial support, educational or training opportunities, employment, and future plans. Participants were also asked about relationships with male partners, including motivation for entering into relationships with male partners, as well as deeper information about relationship challenges, gender roles, conflict management, reasons for conflict or arguments in relationships, ways in which male partners were abusive (e.g., physical incidents, sexual coercion and violence, controlling behaviors, manipulation, and isolation), and any other experiences of IPV. To ensure that the semi-structured interview instruments were appropriate and sensitive to the target population, interview protocols were piloted with five participants. This allowed researchers to find what worked well and what needed to be refined prior to conducting the study interviews.

### 2.4. Analysis

All qualitative interviews were recorded, transcribed, and translated into English by the bilingual (Yoruba-English) facilitators. The coding scheme was based on the interview protocols, adding open codes from emergent themes identified in the interviews. The types of codes included in the analysis focused on economic situations and factors associated with IPV. Economic abuse, which emerged as a common form of IPV across interviews, was also added as a code. Consensus on coding and modifications to the code book occurred during ongoing team meetings. Once finalized, the coding and analysis was conducted using a thematic analysis approach. All interviews were independently coded by two investigators, compared for agreement, and finalized. Additions of new codes or changes in code definitions were determined via consensus among the research team. No new codes emerged after two-thirds of the interviews were coded, suggesting content saturation was achieved.

## 3. Results

A total of 25 women completed the interviews. On average, participants were 21 years old, most had children (68%) and reported to be cohabitating with a male partner (56%), and 48% had less than a secondary education level (Table 1).

Many women reported economic vulnerability as a result of gender inequities in opportunities for education, training, and work. Women commonly reported family challenges to financially support them to continue their education and an unequal division of household labor as being primary factors contributing to reduced opportunities for education and work. These gender and economic inequities precipitated women relying on male partners for financial support. Women reported male partners controlling household finances and denying them adequate allowance to purchase food for the household, including food for the children. Food insecurity due to economic abuse was a common theme reported by the women. Women’s IPV experiences, including sexual and pregnancy coercion, increased their risk for unintended pregnancy, and exacerbated economic vulnerability and ongoing economic abuse. Financial dependence on a male partner and economic abuse made it challenging for women to leave abusive relationships. These various themes and representative quotes from participants are presented below.

### 3.1. Gender Inequities Exacerbate Economic Vulnerability

#### 3.1.1. Inequitable Division of Household Chores

Economic vulnerability among young women in the study was exacerbated by inequitable gender norms. This was noted in the unequal division of household chores. Young women reported being left with a greater burden of the household chores compared to boys. Many also reported that their family believed household chores to be the responsibility of young women in the family.

There are more opportunities for (educating) boys than girls. … Boys are usually left from doing house chores as well, but a girl must do all chores before leaving the house.

ID 8

Boys have better opportunities (to complete their education) than girls … The family expects that the girl child should be able to do house chores, boys are sometimes excluded.

ID 9

#### 3.1.2. Inequitable Financial Support for Girls’ Education and Training

Young women in the study also spoke about not having family or community support to complete their education. Girls reported that families would often prioritize the use of limited financial resources to educate boys rather than girls or young women. Participants indicated that some family and community members believed it is not important to educate women, particularly when there are limited employment opportunities for educated women in the labor market.

Girls are treated differently at home, in the society and everywhere. If I were a boy, even considering that I have a child, they would have supported me to do my ‘freedom’ (completion and graduation from vocational training). They will consider that I am a man and will quickly do it for me. The reason why there is delay in my ‘freedom’ is because I am a girl.

ID 20

People treat girls differently from boys. In my case, when I ask my father for something he will not give me most times, but I have a younger brother that gets anything he wants. The society give more to boys than girls, especially in terms of education. People believe that a girl will end her life in the kitchen no matter her level of education. For this reason, they tend to invest more in boys’ education. The society believes a girl should be good with house chores, neat, hardworking, respectful, jovial.

ID 25

#### 3.1.3. Inequities in Education and Work Opportunities

Gender-based inequities in education and work opportunities also exacerbated economic vulnerability among the participants. Participants discussed the lack of job opportunities for educated women that pay well, and the belief that women cannot do some jobs (e.g., be a mechanic or do security work).

Boys have more opportunities to make money than girls … (including) jobs that bring a lot of money.

ID 14

Boys have better opportunities than girls, there are some jobs that a boy can do that a girl cannot do. Also, sometimes some jobs are left solely for boys.

ID 9

### 3.2. Financial Dependence on Male Partnerships

Without sufficient opportunities for education and employment, young women reported seeking relationships with male partners for financial security. Participants often reported that they were dependent on financial support from a male partner. For one participant, accepting money from a male meant that she could pay her school fees and complete her studies, but at the cost of entering into a relationship with him.

Girls sometimes get favor from men. Some men will help you not necessarily because they are kind, but because they want to have a relationship with you.

ID 11

He told my mother and me to contact him anytime I have a financial challenge in school that he was ready to help. After few months it was time to register for WAEC (standardized exam taken by students in order to graduate from secondary education). My parents couldn’t come up with the payment, so I was chased out of school for non-payment of government levy and other levies that cost NGN 5500 (about USD 15). I was at home for one week. One day I was searching my phone and I saw his number. I called him that I needed his help since he had promised to help me financially when I have a need for my education. He asked me to come and meet him and he gave me the money. I paid the fees with the money and I appreciated him. (This participant was subsequently pressured into having an unwanted relationship with the man who provided funds for her education to clear her “debt” and later became pregnant).

ID 25

### 3.3. Economic Abuse

#### 3.3.1. Experiencing Food Deprivation

Economic vulnerability and financial dependence on a male partner precipitated economic abuse and other forms of IPV. Economic abuse was reported as the male partner’s control over household finances and food deprivation. Women reported that male partners controlled household expenditures for food, and would often withhold money for household food.

The times that we don’t have food is more common. When my husband was jobless, we would eat twice … . Now that he has a job, he doesn’t give money for feeding. We eat whatever we can in a day, even if it is once.

ID 18

He doesn’t buy food in the house; I figure out how we eat. I wasn’t working then but I find a way to manage. If he ever gives money for food, it was never enough. He can drop NGN 300 (USD 0.80) or NGN 200 (USD 0.55). He only does this when he is happy. If he ever gives me NGN 500 (USD 1.38) for feeding, it may be all for the next one week.

ID 21

When I was with my husband, there are times he gives me money for food and I sort things out at other times. Sometimes he will not give me money for feeding and he will tell me the kind of food I should prepare. I used to refuse him any special food at that time, I only cook what I can afford from the money that my father, mother, and grandma give me.

ID 13

Money causes fight a lot. He will ask me to cook for him without dropping any money or dropping very little. If I complain that what he gave is too small, he will collect it back and that is all. If I don’t cook the food, it is trouble. If he gave instruction that I should cook rice and boiled egg and I don’t add egg because the money I have is not enough, that is another trouble.

ID 21

He may ask me to cook when he didn’t give me any money. If he returns and didn’t get food, he may be angry. He can start to shout on me and get really angry. If I explain that I don’t have money, he will insist I have money. He can start to shout and threaten to lock me out if he goes out to eat. Truly, he will lock me out and I will go and sleep in his mother’s house. His mother’s house is at (a nearby community). His mother will follow me in the morning to come and settle the quarrel.

ID 14

#### 3.3.2. Sexual Favors in Exchange for Financial Support

Economic abuse was also reported as sexual favors a male partner expected in return for any money, gifts, or food allowance he provided to the young woman. Participants reported that the male partner expected sex in exchange for financial support, and would withhold money unless he received sexual favors. This exchange of sex and financial support was reported by both women who were single, as well as those who were in committed relationships.

I had a man that will not give me money unless he sleeps with me … We quarrel about money a lot because if I ask him for money, he will not give me. If I need money from him, I ensure I get it when he wants to have sex with me, because that is the only way I am sure he will give me without a blink.

ID 11

If I refused to have sex with him, he will not give me and the child feeding allowance. Also, if you have sex with him today, that does not stop him from beating you tomorrow. Sometimes, when he wants to have sex with me, he will ask me for how much I want. Many times, I refuse his offer and tell him I am not a prostitute. Other times when I need money badly, I will (have sex) to collect the money from him or ask him to buy me Maltina (a beverage) and milk.

ID 21

Financial dependence has turned some girls into prostitutes, some have contracted HIV.

ID 5

### 3.4. Pregnancy and IPV

Financial dependence on a male partner and the male’s expectation of sex in exchange for financial support were also reported to place young women at risk of an unwanted pregnancy. Young women in the study reported pregnancy coercion as a form of IPV. For some young women, the male partner intentionally got them pregnant or pressured them into becoming pregnant by not allowing them to use birth control.

I don’t know his thought. (I think) he intentionally got me pregnant because of the ways boys were always around me.

ID 21

No, I didn’t let him know before I took the method (birth control method). If I told him, he wouldn’t have allowed me.

ID 16

He used to pressure me to get pregnant and that was how the pregnancy happened.

ID 13

IPV was reported to worsen during pregnancy for some of the women, specifically physical violence and other threats. While many young women reported pregnancy coercion, one woman also reported her partner attempted to force her to have an abortion.

He beat me when I was about seven months pregnant. I was tired and he asked me to cook for him. I told him that I was sick and couldn’t cook the food. My neighbor came out and appealed to me to cook the food. When I got up to cook the food, he stopped me that he doesn’t want me to cook for him anymore. He went ahead to start cooking. When he came back to the room, he punched me on my stomach. The beating was so much. I angrily went to remove what he was cooking and poured it on him. This kind of incidents when he beats me was a common thing in our relationship then. I got used to it at a point. His eldest sibling came and intervened. He appealed that we should settle and that I should forgive his attitude.

ID 21

When I suspected I was pregnant, I went to a chemist in front of my shop for pregnancy test and I told my boyfriend. He later took me to a hospital and I overheard when he was telling the doctor about abortion, I jumped up from the bed and ran away. After that he poisoned my drink, not knowing I didn’t take it. Few days later, he called me to ask if I have seen any blood. I told him no and since then I will not eat any food or drink he gives me. That was how we started real fight and he really abused me.

ID 5

### 3.5. Pregnancy and Diminished Financial Support from a Male Partner

Many of the women stated that once they became pregnant, had the child or moved in with the male partner, the financial support they initially received in the beginning of the relationship diminished significantly. This placed women in an even more economically vulnerable position and at risk of economic abuse.

To outsiders, he is a good person but not at home. He was good to me too before I moved to his house. You can’t know the character of a man when you are still dating or don’t have a child, until you are in his house … Then, he took me out, gave me money and bought things for me. But nothing of such happens again now. (The male partner was having an affair with another woman the entire time the participant was dating him. The participant later got pregnant and was no longer supported financially by the male partner).

ID 18

We lived together for one year and six months. When we were together, he took care of me, he buys things for me. He was very caring for me in pregnancy till I was delivered of my child.

ID 21

Women who were either pregnant or caring for a child were particularly dependent on their male partner for money to purchase food. Often, women reported that the male partner would only provide a very small amount of money for food or not provide anything at all.

Some of the challenges I face is money. When I ask my husband for money to buy things for my daughter, he will not give me … When our daughter has not eaten and there is no food at home to give her, my husband will keep telling me to breastfeed her. This is a girl that is not satisfied with breast milk anymore.

ID 12

I wasn’t working initially, my pregnancy period and after. So, I was solely dependent on him for money. If he doesn’t drop money, I will not eat. When I was pregnant, I will be at home hungry and he will be giving people money.

ID 10

When I was pregnant, I called him that there was no money to feed. That time, I only eat once a day because no one will feed me. My mother didn’t have money then to even support me. Her shop was empty. Sometimes, my siblings will not go to school because there was no money to give them. If I called him that I needed money, he will just tell me he was coming and he will not. One day my friend told me he saw him in the next street, so I went there to meet him. I asked him why he didn’t send money to me. He gave me NGN 500 (USD 1.20). I told him it was not enough but he ignored me.

ID 25

### 3.6. Pregnancy and Childcare Inhibiting Economic Opportunities

Young women in the study spoke about reduced opportunities to take final exams, continue their education, or complete an apprenticeship because of a pregnancy or the need to care for their child. Young women discussed not being able to move around for a job while caring for a child or that employers did not want to hire a pregnant woman. Pregnancy and caring for a child increased economic vulnerability, and contributed to the difficulty women faced in becoming financially independent from a male partner.

Firstly, I am not free among my friends, because I will be the only one carrying a baby when we are all together. I hide from my friends. It affected my education. If I didn’t have a child, I would have written my exam again. Thirdly, it affected my finance. I can’t move around to get a job because I can’t leave a baby.

ID 25

If I was a boy, I would have more opportunity to ‘hustle’ and work for money. Boys are not disturbed by carrying children around.

ID 12

This pregnancy has cost me a lot. First, when I wanted to quit my current job, I was told employment cannot be given to a pregnant person. I also wanted to rewrite my secondary school exam, but I was told to wait till after my delivery. I wish to go back to school too but I was told to wait ‘til I am delivered of the baby.

ID 9

I ended my education because of the pregnancy. The pregnancy led to a quarrel between my eldest sibling and me ‘til today. He stopped being involved in my life since I got pregnant. My eldest sibling is a graduate and dislikes an uneducated person. He has not seen my husband before. He is not aware that I am back to my mother’s place. He told me that the only thing that will make him involved with me again is if I am graduating from my apprenticeship or go back to school.

ID 16

## 4. Discussion

This study describes how gender inequities create economic vulnerabilities among young women in our sample, that, in turn, may influence the ways in which young women experience or are at risk for economic abuse and other forms of IPV. The findings from this study point to gender norms that devalue young women’s education and training, and limit employment opportunities for young women, all of which played a significant role in creating economic vulnerability among the young women in our sample. The findings highlight how economic vulnerability appears to promote financial dependence on a male partner for basic needs, and precipitates vulnerability to economic abuse and other instances of IPV. Our findings suggest that IPV in the form of coerced sex leading to unintended pregnancy further restricts young women’s opportunities to finish their education or vocational training and to find employment. Our findings showcase economic abuse in the form of male partners expecting sex in exchange for financial support, and male partners controlling household income. Building upon the existing literature on economic abuse, our findings highlight food deprivation as a form of economic abuse, resulting in substantial food insecurity among women and their children.

The findings from this study highlight the intersection of economic abuse and food insecurity among this sample of young women and their children in Nigeria who were already experiencing high levels of poverty [9]. Previous studies have documented the consequences of economic abuse to be wide-reaching, and include increased risk of maternal morbidity/mortality, sexual exploitation, HIV (if women engage in sexual transactions with males in return for money), and negative impact on the mental health of women and their children [7,23,28]. Our findings build upon the few but expanding number of studies to understand food insecurity in the context of economic abuse, particularly in Nigeria. In our sample, economic abuse reported by young women typically involved male control over household financial decision-making, as well as deprivation of sufficient money for household food. The connection noted in our study between economic abuse and substantial food insecurity correlates with findings from the UN multi-country study on men and violence, which found that women in Asia and the Pacific who experience economic abuse have 1.69-fold increased odds of experiencing food insecurity, particularly when male partners withhold money to pay for food expenses [29]. Further quantitative research is needed to better contextualize the prevalence and extent of economic abuse and food insecurity among women and their children, particularly in settings where women already experience high levels of IPV and economic vulnerability.

Our findings, related to the primary contributing factors of economic vulnerability among young women, are similar to those found in previous work. Multiple studies have documented the lack of prioritizing household resources to support young women’s education [27,30,31]. Notably, young women in our study were very aware of and able to describe the specific factors that limited their ability to complete their education, including the lack of funds for their education, the disproportionate burden of household labor put on young women, unplanned pregnancy, and the overall prioritization of male siblings to receive any type of household resources and investment. Our findings on the lack of financial support for young women to finish their education contribute to a better understanding of the barriers many young women may face in completing their education or vocational training, and in finding work opportunities that allow them to be financially independent and empowered in financial decision-making for them and their families. Such findings are aligned with previous work, and build upon the existing literature to highlight the intersection of structural and social factors that place many young women at risk for experiencing economic vulnerability [5,20,21,27,32].

Young women in our sample reported reproductive coercion and unintended pregnancy that further worsened their experiences of economic vulnerability and ongoing IPV. Many young women reported that reproductive coercion (noted as coerced sex, contraceptive sabotage, and forced pregnancy) was largely a way for their male partner to assert further control in the relationship. Our findings linking reproductive coercion, IPV, and unintended pregnancy are consistent with previous research. In a systematic review of reproductive coercion (defined as pregnancy coercion, birth control disapproval, birth control sabotage, and abortion coercion), the majority of studies found that women who experienced IPV and controlling behavior from the male partner were at an increased risk for experiencing birth control sabotage and pregnancy coercion [33]. Reproductive coercion is also associated with an increased risk of unintended pregnancy [34]. This study is one of few that explores reproductive coercion in the context of economic abuse in Nigeria. As indicated from our findings, pregnancy or caring for children further contributes to a woman’s financial dependence on a male partner and risk for economic abuse.

Our study also sheds light on a form of economic abuse, where young women reported that financial resources from male partners were often only provided with the expectation of sex in return, regardless of the type of relationship (married, dating, or intimate). These findings align with previous studies in sub-Saharan Africa on transactional sex among adolescent and young women [20,35]. Women’s engagement in transactional sex for the purpose of improving their social status and gaining access to material goods has been documented across several studies and is called various names, including “milking the cow” in Mozambique [36], “skinning the goat” in Tanzania [37], and “de-toothing” in Uganda [38,39]. Across these different studies, the authors describe women’s strategies to extract resources (i.e., clothes, phones, and other wanted materials) from a male partner. Different from these previous studies, where young women enter into relationships for the purpose of gaining material goods such as clothes or other gifts, the participants in our study described exchanging sex as a mechanism to meet their basic needs, such as obtaining food for themselves and their children. Among the young women in our study, the need for money to pay for food and other basic needs may place young women at greater risk of financial dependence on a male partner, which then further increases the potential for unintended pregnancy, as well as ongoing IPV and economic abuse.

A limitation of this study is that our findings are specific to this urban population of women reporting a history of IPV recruited from low-income neighborhoods in Ibadan, Nigeria. Future qualitative and quantitative studies are needed to understand the connection between economic abuse and economic vulnerability from a broader population of young women to better understand the experiences of women from other backgrounds and communities. In addition, future quantitative studies are needed to assess the prevalence of economic abuse, and its associations with food insecurity as well as other negative consequences.

## 5. Conclusions

This study highlights economic vulnerability, financial reliance on male partners, experiences of economic abuse, and other forms of IPV among a sample of young women with a history of IPV from low-income neighborhoods in Ibadan, Nigeria. The consequences of economic abuse and other forms of IPV, including unintended pregnancies, furthered women’s economic vulnerability and financial reliance on their male partners. This study’s findings suggest the need for implementing programming and policy efforts to remove barriers for girls and young women to achieve an education and to gain employment opportunities. Future research and programmatic work are also needed to better understand and address the intersection of food insecurity and economic abuse affecting women and their children. Our findings also suggest that programs promoting food security among women and children may also need to consider economic abuse and IPV as factors contributing to high levels of food insecurity among this population.

## Figures and Tables

**Table 1.** Socio-demographic characteristics of participants.

Characteristic	Frequency (%) n = 25
Age (years)	
18–19	6 (24)
20–22	11 (44)
23–25	6 (24)
26–28	2 (8)
Level of Education	
No Formal Education	1 (4)
Primary (Elementary)	3 (12)
Incomplete Secondary	8 (32)
Secondary (Middle School)	10 (40)
University/College	3 (12)
Marital Status	
Single/Never Married	3 (12)
Cohabiting and Not Married	14 (56)
Married/Common Law	8 (32)
Have Children	
Yes	17 (68)
No	8 (32)

## Data Availability

Data available on request from the authors.
